# Sodium Hyaluronate in the Treatment of Dry Eye Syndrome: A Systematic Review and Meta-Analysis

**DOI:** 10.1038/s41598-017-08534-5

**Published:** 2017-08-21

**Authors:** Bryan Chin Hou Ang, James Jie Sng, Priscilla Xin Hui Wang, Hla Myint Htoon, Louis Hak Tien Tong

**Affiliations:** 1 National Healthcare Group Eye Institute, Tan Tock Seng Hospital, Singapore, Singapore; 2 Singapore National Eye Centre, Singapore, Singapore; 3 Singapore Eye Research Institute, Singapore, Singapore

## Abstract

This systematic review and meta-analysis compares sodium hyaluronate (HY) with non-HY based artificial tears in the treatment of dry eye syndrome. A literature search for clinical trials comparing HY against non-HY preparations was conducted across PubMed, Cochrane Central Register of Controlled Trials and Scopus databases from inception up to May 2016. Majority of the 18 studies selected for review showed superiority of HY in improving ocular staining and symptoms. Randomized controlled trials (RCTs) examining Schirmer’s I (SH) and tear breakup time (TBUT) underwent further meta-analyses with calculation of pooled standardized mean differences (SMDs) with 95% confidence intervals (CIs). 7 RCTs including 383 eyes randomized to HY and 596 eyes to non-HY preparations underwent meta-analysis for SH. 9 RCTs including 458 eyes randomized to HY and 651 eyes to non-HY preparations underwent meta-analysis for TBUT. By fixed-effects modelling, HY demonstrated greater improvement of SH compared to non-HY preparations (SMD, 0.238; 95% CI, 0.107 to 0.369; p < 0.001). By random-effects modelling, HY demonstrated less improvement of TBUT (SMD, −0.566; 95% CI, −1.099 to −0.0336; p = 0.037). In summary, neither preparation was shown to be consistently superior across all outcome measures. The difference in effect between preparations on SH and TBUT was not clinically significant.

## Introduction

The International Dry Eye Workshop^1^ defines dry eye syndrome (DES) as a multifactorial heterogeneous disease of the tear film and ocular surface that results in discomfort, visual acuity disturbance and tear film instability. It arises when there is disruption to the lid surface and function, cornea sensation, blink reflex, tear film production and stability, tear composition and drainage^2, 3^ leading to complications such as persistent corneal epitheliopathy and corneal infection. DES affects nearly 3.23 million women, 1.68 million men and 4.91 million Americans 50 years and older^4^.

A wide range of treatment options is available in the management of DES, including topical lubricants or artificial tear substitutes, ointments or gels, topical secretagogues, anti-inflammatory therapy, biologic tear substitutes, punctal occlusion, moisture chamber goggles and surgery. Artificial tears arguably provide the most affordable, immediate and least invasive form of relief. Previous generations include integrated natural polymers (such as cellulose derivatives) and synthetic polymers [(such as polyvinyl alcohol (PVA) and hydroxypropylguar (HP Guar)].

In recent decades, sodium hyaluronate has emerged as an option in artificial tear therapy. It owes its efficacy to hyaluronic acid (HY), a naturally occurring linear biopolymer consisting of repeating disaccharide units of N-acetyl-D-glucosamine and sodium-D-glucoronate. HY is widely used today and has been shown to result in both subjective and objective improvement of DES. A recent meta-analysis by Kong X. *et al*.^5^ compared the effect of HY and non-HY preparations in symptomatic relief of subjects and could not demonstrate superiority of one preparation over the other. However, the comparative efficacy of both artificial tears with regard to all other common outcome measures of DES remains unclear.

The aim of this systematic review and meta-analyses is to compare the efficacy of sodium hyaluronate against non-HY based preparations in the treatment of DES.

## Results

### Literature Retrieval Results

**Figure 1:**
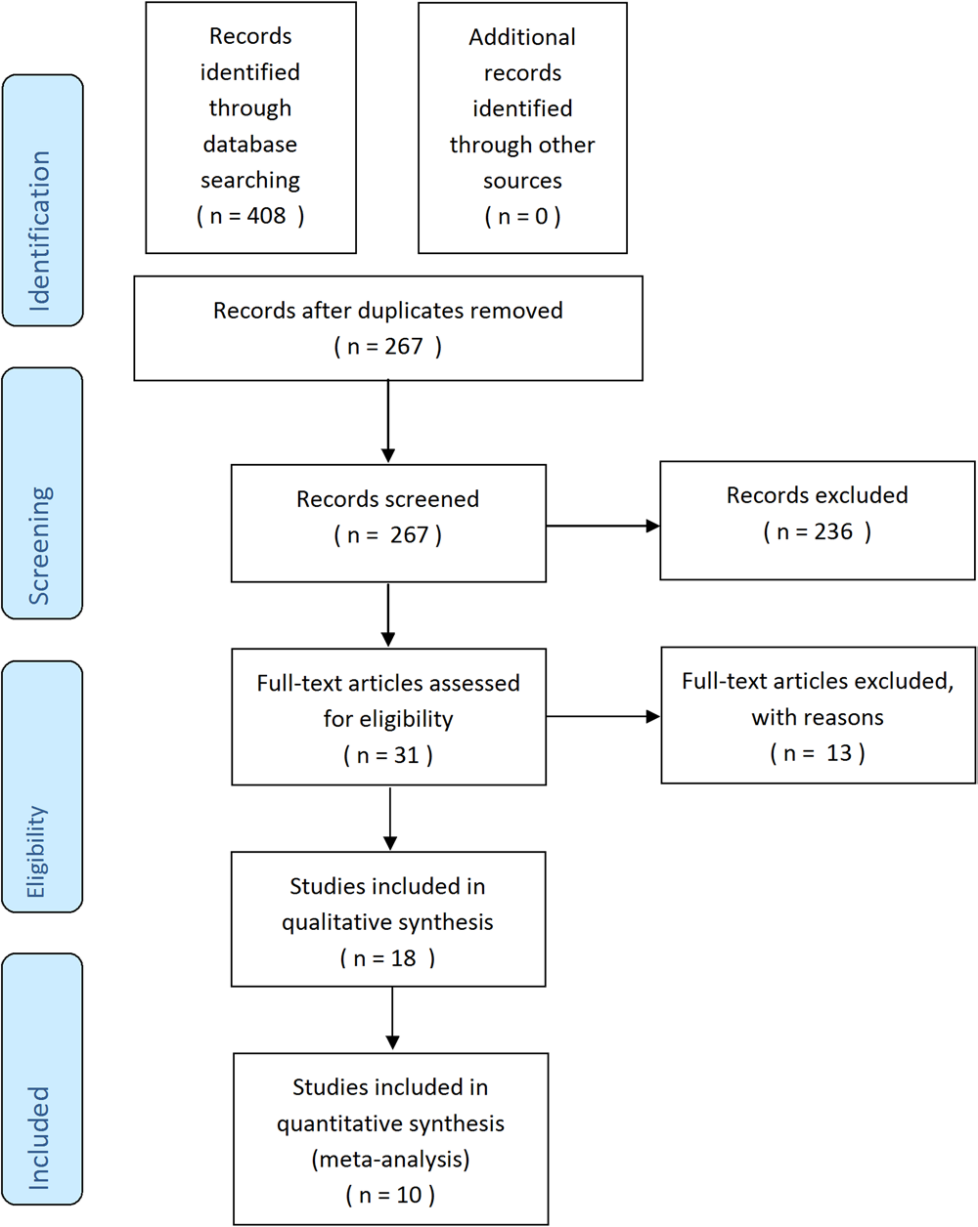
Study Selection Process and Results. Adapted from: PLoS Medicine (OPEN ACCESS) Moher D, Liberati A, Tetzlaff J, Altman DG, The PRISMA Group (2009). Preferred Reporting Items for Systematic Reviews and Meta-Analyses: The PRISMA Statement. PLoS Med 6(7): e1000097. doi:10.1371/journal.pmed1000097.

The initial literature search generated a total of 408 articles. 141 duplicates were removed. Based on the inclusion criteria, 247 articles were deemed unsuitable. Table 1 details the reasons for exclusion of studies. Of the remaining 20 studies, the full-text articles of 18 studies^6–23^ were successfully retrieved and included in the systematic review. Of these, 10 were RCTs which reported SH I and/or TBUT results^6, 7, 9–11, 14, 18, 19, 21, 22^ and underwent further meta-analyses. Figure 1 summarizes the study selection process.

**Table 1:** Reasons for Exclusion of Studies.

S/N	Reason for Exclusion	No. of Studies
1	Not journal article (e.g. conference abstracts, etc)	5
2	Not ocular surface-related study	12
3	Not clinical, *in vivo* trial	66
3	Not HY-related study	18
4	Not conducted in human subjects	25
5	Not interventional	2
6	Not published in English	14
7	No HY-only study arm	35
8	No Non-HY only study arm	31
9	Non-HY only study arm includes an active compound: diquafosol (5), rebamipide (1), TSP (1), trehalose (1), prednisolone (2), others: (9)	19
10	Less than 1 week follow-up of subjects	11
11	Subjects on long-term eyedrops (e.g. glaucoma medications) or contact lenses	3
12	Employs concurrent fellow-eye comparison	2
13	Conducted in post-refractive surgery patients	4
	Total	247

HY = Hyaluronic Acid; TSP = Tamarindus indica seed polysaccharide.

### Characteristics of Studies

A total of 18 studies, published between 1987 to 2012, were included in this review. Only 1 study was conducted in Asia, while the rest were conducted in Europe and the United States of America. The majority were parallel studies (12 studies) and the others were crossover studies (6 studies). All the parallel studies and 4 of the 6 crossover studies were randomized (crossover studies were randomized to which eyedrop subjects started with). 16 studies were single or double-masked, while the remaining 2 did not indicate if masking was carried out. The studies recruited subjects with DES of varying severities and followed them up to between 14 to 90 days. The preparations of HY ranged from 0.1% to 0.4% and most of the control eyedrops were MC-based (7 studies) or PVA-based (5 studies). Key study characteristics are summarized in Table 2.

**Table 2:** Summary of Key Characteristics of Studies.

S/N	Study	Location	Study Design	Randomization	Masking	Follow Up Duration	Mean Age of Subjects	Severity of DES of Subjects	% of HY	Eyedrop/s of Selected Control Arm/s
1	Baeyens *et al*.^6^	United Kingdom, France	Parallel	●	Double	84 days	59.3	Mild to moderate DES	0.18	0.3% Carbomer, Saline
2	Baudouin *et al*.^7^	France	Parallel	●	Single (investigator)	35 days	57	Severe DES excluded	0.18	Osmoprotective-CMC
3	McCann *et al*.^8^	United Kingdom	Parallel	●	Single (investigator)	90 days	43.4	Mild to moderate DES	0.15	HPMC
4	Lee *et al*.^9^	Korea	Parallel	●	Single (observer)	8 weeks	38	Mild to moderate DES	0.1	0.5% CMC
5	Benellli *et al*.^10^	Italy	Parallel	●	Single (investigator)	1 month	Not provided	ODSI-II value between 30 and 60 and SH < 7mm after 5 min	0.2	0.5% CMC, 0.18% HP Guar
6	Sanchez *et al*.^11^	Spain	Parallel	●	Single (observer)	30 days	71.8	Sjögren’s syndrome or primary DES	0.15	0.5% Carmellose
7	Vogel *et al*.^12^	United States of America	Parallel	●	Double	14 days	61.5	Not provided	0.18	Vehicle (identical to study drug but lacking HY)
8	Johnson *et al*., 2008	United Kingdom	Parallel	●	Double	30 days	Median = 38 (range 21–64)	Moderate DES	0.18	0.3% Carbomer
9	Brignole *et al*.^14^	France	Parallel	●	Single (observer)	56 days	63.3	Moderate DES (Sjögren’s syndrome or primary DES)	0.18	1% CMC
10	Aragona *et al*.^15^	Italy	Parallel	●	Double	3 months	50.5	Moderate to severe DES	0.15	Saline/0.9% Sodium Chloride
11	Benitez *et al*.^16^	Spain	Crossover	○ (single group study only)	Nil	2 weeks	57	Moderate to severe DES	0.18	1.4% PVA with BAK
12	MacDonald *et al*., 2002	United Kingdom	Crossover	● (for which eyedrop to start with)	Double	4 weeks	58.8	Severe DES	0.1	1.4% PVA
13	Iester *et al*.^18^	Italy	Parallel	●	Nil	60 days	54.2	Moderate to severe DES	0.4	HPMC
14	Condon *et al*.^19^	United Kingdom	Crossover	● (for which eyedrop to start with)	Double	28 days	60	Severe DES	0.1	0.9% Saline
15	Sand *et al*.^20^	Denmark	Crossover	○ (single group study only)	Double	14 days	Median = 60.5 (range 42–78)	Severe DES	0.1, 0.2	Placebo (buffer solution in which HY was dissolved)
16	Laflamme *et al*.^21^	Canada	Crossover	● (for which eyedrop to start with)	Nil	8 weeks	58	Severe DES	0.1	1.4% PVA
17	Nelson *et al*.^22^	United States of America	Parallel	●	Double	56 days	59.4	Moderately severe DES	0.1	1.4% PVA with 0.5% Chlorobutanol
18	Limberg *et al*.^23^	United States of America	Crossover	● (for which eyedrop to start with)	Double	2 weeks	65	SH < 11mm after 5 min, complaints attributable to KCS, RB staining of cornea or conjunctiva, decreased marginal tear strip	0.1	1% PVA and polyethylene glycol

DES = Dry Eye Symptoms; HY = Hyaluronic Acid; PVA = Polyvinyl Alcohol; BAK = Benzalkonium Chloride; HPMC = 0.3% Hydroxypropylmethylcellulose +0.1% Dextran 70; SH = Schirmer’s Test.

Outcome measures examined across all studies are summarized in Table 3. TBUT, SH I, ocular staining and symptoms were the 4 most commonly studied outcome measures.

**Table 3:** Outcome Measures Across Studies.

S/N	Study	TBUT	SH I	Ocular Staining (Cornea or Conjunctiva, Scoring System)	Symptoms (Scoring/Question-naire)	Safety	Tear Osm	Conjunctival Impression with Flow Cytometry	Conjunctival Impression without Flow Cytometry	VA	Tear Meni-scus	NIT-BUT	Corneal Topogr-aphy	TER	TTR	TFS	TPH	Corn-eal AF	TCR	TFI	TFP	Corneal Sensiti-vity
1	Baeyens *et al*.^6^	●	●	● (not specified cornea/conjunctiva; Lissamine Green - score 0–12; Sodium Fluorescein - score 0–7)	● (VAS, frequency score, impact on ADL score)	●	○	○	○	●	○	○	○	○	○	○	○	○	○	○	○	○
2	Baudouin *et al*.^7^	●	●	● (cornea, conjunctiva; Oxford Scheme; Sodium Fluorescein)	● (OSDI)	●	●	○	○	○	○	○	○	○	○	○	○	○	○	○	○	○
3	McCann *et al*.^8^	○	○	● (cornea; Oxford Scheme; Sodium Fluorescein)	● (McMonnies, SANDE)	○	●	○	○	○	○	●	○	●	●	●	○	○	○	○	○	○
4	Lee *et al*.^9^	●	○	● (cornea, conjunctiva; Sodium Fluorescein)	●	●	○	○	○	○	○	○	○	○	○	○	○	○	○	○	○	○
5	Benellli *et al*.^10^	●	●	● (cornea, conjunctiva; Sodium Fluorescein)	○	○	●	○	○	●	○	○	●	○	○	○	○	○	○	○	○	○
6	Sanchez *et al*.^11^	●	○	● (cornea; Oxford Scheme; Sodium Fluorescein)	○	○	○	●	○	○	○	○	○	○	○	○	○	○	○	○	○	○
7	Vogel *et al*.^12^	○	●	● (cornea, conjunctiva; Sodium Fluorescein)	● (VAS, GFS, GIS, ADL)	●	○	○	○	○	○	○	○	○	○	○	○	○	○	○	○	○
8	Johnson *et al*., 2008	●	○	● (cornea, conjunctiva; Oxford Scheme; 2% Sodium Fluorescein)	● (OCI)	○	○	○	○	○	○	●	○	○	○	○	○	○	○	○	○	○
9	Brignole *et al*.^14^	●	○	● (cornea, conjunctiva; Sodium Fluorescein)	● (McMonnies, VAS)	●	○	●	○	○	○	○	●	○	○	○	●	○	○	○	○	○
10	Aragona *et al*.^15^	●	○	● (not specified cornea/conjunctiva; Rose Bengal, Sodium Fluorescein)	● (VARS)	●	○	○	●	○	○	○	○	○	○	○	○	○	○	○	○	○
11	Benitez *et al*.^16^	○	○	○	○	○	○	○	○	○	○	○	○	○	○	○	○	●	○	○	○	○
12	Mac-Donald *et al*., 2002	●	●	● (cornea, conjunctiva; Rose Bengal)	● (VAS)	●	○	○	○	○	●	○	○	○	○	○	○	○	●	●	○	○
13	Iester *et al*.^18^	●	●	● (cornea, conjunctiva; Rose Bengal, Sodium Fluorescein)	●	○	●	○	●	○	○	○	○	○	○	○	○	○	○	○	●	○
14	Condon *et al*.^19^	○	●	● (cornea, conjunctiva; Rose Bengal)	●	●	○	○	○	○	○	○	○	○	○	○	○	○	○	○	○	○
15	Sand *et al*.^20^	●	●	● (not specified cornea/conjunctiva; Rose Bengal)	● (VAS)	○	○	○	○	○	○	○	○	○	○	○	○	○	○	○	○	●
16	Laflamme *et al*.^21^	●	●	● (cornea; Sodium Fluorescein)	● (VAS)	○	○	○	○	○	●	○	○	○	○	○	○	○	○	○	○	○
17	Nelson *et al*.^22^	●	●	● (cornea, conjunctiva; Rose Bengal)	● (VAS)	○	●	○	●	●	○	○	○	○	○	○	○	○	○	○	○	○
18	Limberg *et al*.^23^	●	●	● (cornea, conjunctiva; Rose Bengal)	●	○	○	○	○	●	●	○	○	○	○	○	○	○	○	○	○	○

ADL = Activities of Daily Living; Corneal AF = Corneal Autofluorescence (with Fluorophotometer); GFS = Global Frequency Score; GIS = Global Intensity Score; NITBUT = Non-invasive Tear Break-up Time; OCI = Ocular Comfort Index; ODSI = Ocular Surface Disease Index; SANDE = Symptom Assessment in Dry Eye; SH I = Schirmer’s Test I (without anaesthesia); TBUT = Tear Break-up Time; TCR = Tear Clearance Rate; TER = Tear Evaporation Rate (Evaporimetry); TFI = Tear Function Index (=Shirmer’s/TCR); TFP = Tear Ferning Pattern Test; TFS = Tear Film Stability (Interferometry); TPH = Tear Prism Height; TTR = Tear Turnover Rate; VA = Visual Acuity; VAS = Visual Analogue Scale; VARS = Visual Analogue Rating Scale; ● = Yes; ○ = No.

### Risk of Bias Assessment

The risk of bias assessment of the 10 RCTs which underwent further meta-analyses are presented in Table 4. For selection bias, the risk for all the studies, with the exception of Condon *et al*.^19^ could not be determined due to the lack of information regarding the randomization sequence process and allocation concealment. 7 of the 10 studies were observer/investigator-masked and assessed to be at low-risk of detection bias. However, only 3 studies blinded study participants. Majority of studies (8 of 10) did not have any missing outcome data, or had missing data which was deemed insignificant for the study to be considered at high-risk of attrition bias. Although we did not have access to detailed study protocols, we assessed all the included studies to be at low-risk of selective reporting bias, given that all the outcomes described in their respective methodology sections were reported in their results.

**Table 4:** Risk of Bias Assessment for Studies Included in Meta-Analysis.

S/N	Study	Selection Bias		Performance Bias		Attrition Bias	Reporting Bias
		Random Sequence Generation	Allocation Concealment	Blinding of Participants and Personnel	Blinding of Outcome Assessment	Incomplete Outcome Data	Selective Reporting
1	Baeyens *et al*.^6^	?	?	−	−	−	−
2	Baudouin *et al*.^7^	?	?	+	−	−	−
3	Lee *et al*.^9^	?	?	+	−	−	−
4	Benellli *et al*.^10^	?	?	+	?	−	−
5	Sanchez *et al*.^11^	?	?	+	−	−	−
6	Brignole *et al*.^14^	?	?	+	−	−	−
7	Iester *et al*.^18^	?	?	+	+	?	−
8	Condon *et al*.^19^	−	−	−	−	−	−
9	Laflamme *et al*.^21^	?	?	+	+	+	−
10	Nelson *et al*.^22^	?	?	−	−	−	−

+ = High Risk; − = Low Risk; ? = Unclear Risk.

### Quantitative Analyses

#### SH I

**Figure 2:**
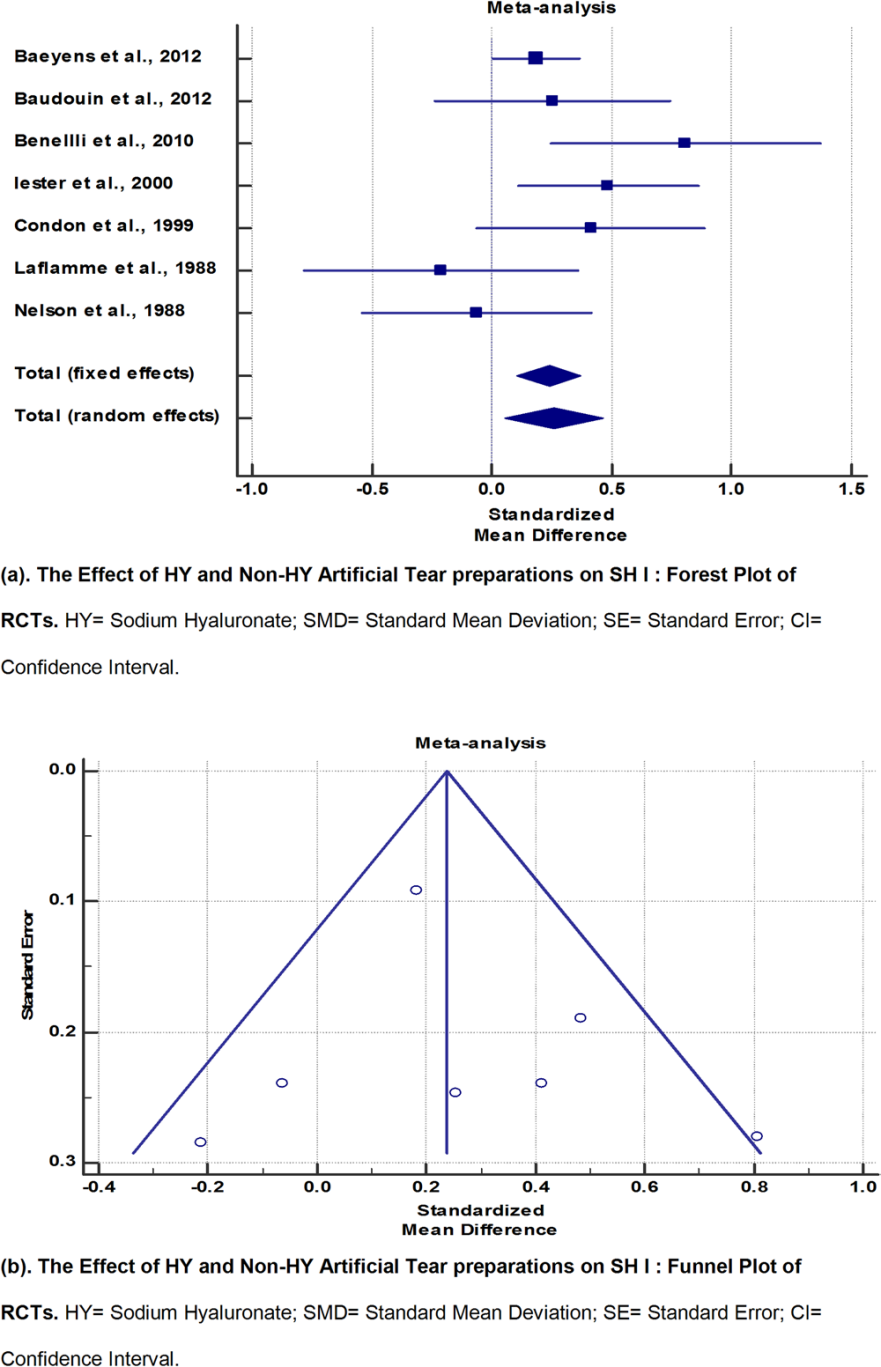
The Effect of HY and Non-HY Artificial Tear preparations on SH I: Forest Plot, and Funnel Plot of RCTs. HY = Sodium Hyaluronate; SMD = Standard Mean Deviation; SE = Standard Error; CI = Confidence Interval.

7 RCTs^6, 7, 10, 18, 19, 21, 22^ were included in the meta-analysis for SH I outcomes (Fig. 2 and Table 5). A pooled total of 383 eyes were randomized to HY preparations and 596 eyes to non-HY preparations. The I^2^ value of 44.25% reflected acceptable heterogeneity, with corresponding symmetry of the funnel plot. By fixed-effects modelling, eyes in the HY group demonstrated greater improvement of SH compared to non-HY preparations (SMD, 0.238; 95% CI, 0.107 to 0.369), with results reaching statistical significance (p < 0.001).

**Table 5:** The Effect of HY and Non-HY Artificial Tear preparations on SH I: Meta-Analytic Data of RCTs.

Study	No. of subjects in HY arm	No. of subjects in Non-HY arm	Total	SMD	SE	95% CI	t	P	Weight (%)	
									Fixed	Random
Baeyens *et al*.^6^	178	374	552	0.183	0.0911	0.00428 to 0.362			53.58	27.53
Baudouin *et al*.^7^	29	37	66	0.253	0.246	−0.238 to 0.745			7.34	11.89
Benellli *et al*.^10^	20	40	60	0.806	0.280	0.245 to 1.367			5.67	9.95
Iester *et al*.^18^	58	55	113	0.484	0.190	0.108 to 0.860			12.36	16.22
Condon *et al*.^19^	34	36	70	0.411	0.239	−0.0657 to 0.888			7.78	12.35
Laflamme *et al*.^21^	24	24	48	−0.212	0.285	−0.785 to 0.361			5.48	9.72
Nelson *et al*.^22^	40	30	70	−0.0620	0.239	−0.539 to 0.415			7.79	12.35
Total (fixed effects)	383	596	979	0.238	0.0667	0.107 to 0.369	3.566	<0.001	100.00	100.00
Total (random effects)	383	596	979	0.262	0.105	0.0566 to 0.467	2.504	0.012	100.00	100.00
Test for heterogeneity										
Q	10.7616									
DF	6									
Significance level	P = 0.0960									
I^2^ (inconsistency)	44.25%									
95% CI for I^2^	0.00 to 76.54									

HY = Sodium Hyaluronate; SMD = Standard Mean Deviation; SE = Standard Error; CI = Confidence Interval.

#### TBUT

**Figure 3:**
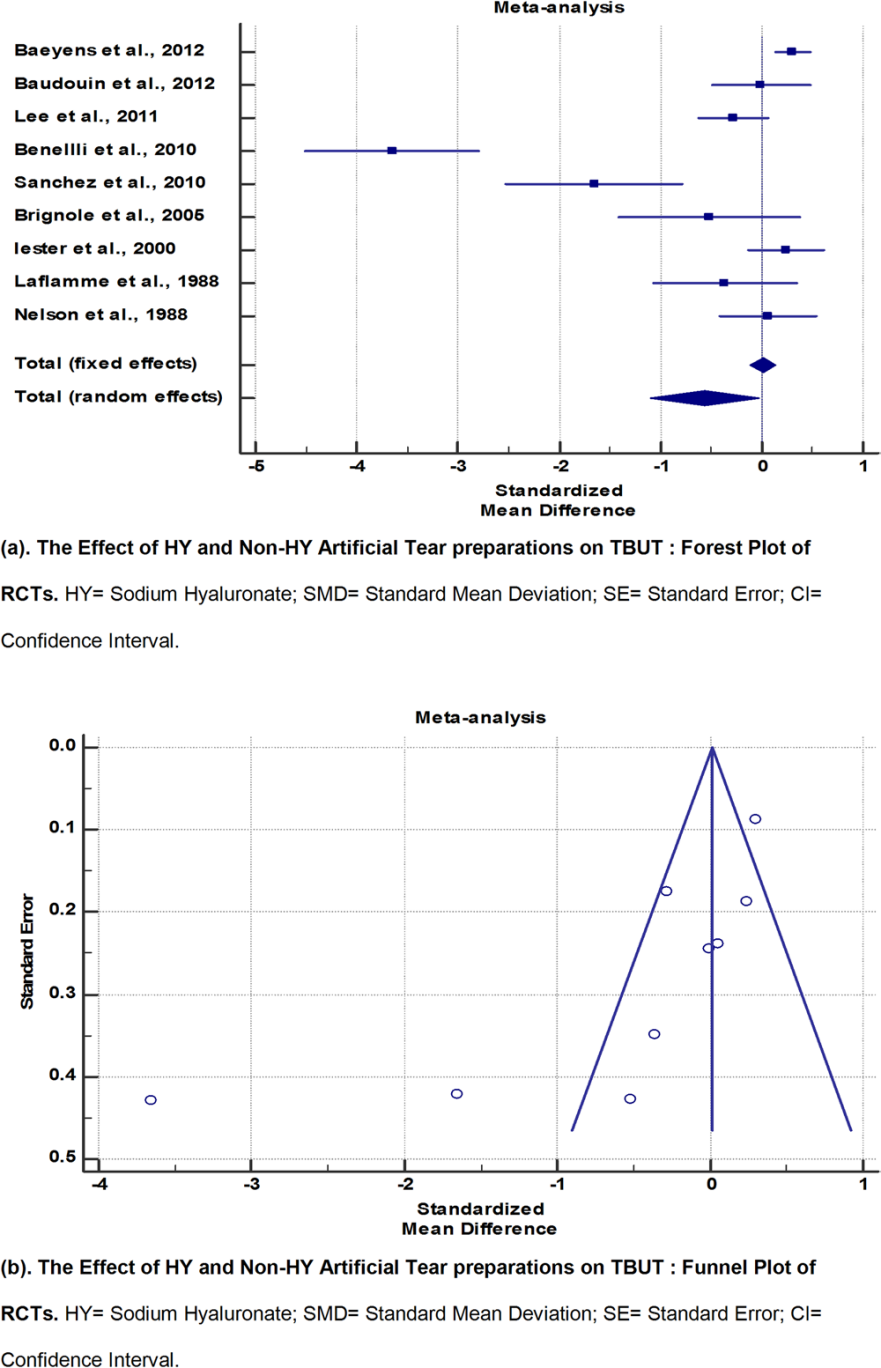
The Effect of HY and Non-HY Artificial Tear preparations on TBUT: Forest Plot and Funnel Plot of RCTs. HY = Sodium Hyaluronate; SMD = Standard Mean Deviation; SE = Standard Error; CI = Confidence Interval.

9 RCTs^6–8, 10, 11, 14, 18, 21, 22^ were were included in the meta-analysis for TBUT outcomes (Fig. 3 and Table 6). A pooled total of 446 eyes were randomized to HY preparations and 651 eyes to non-HY preparations. TheI^2^ value of 92.49% reflected significant heterogeneity, with corresponding skewing of the funnel plot. By random-effects modelling, eyes in the HY group demonstrated less improvement of TBUT compared to eyes in the non-HY group (SMD, −0.566; 95% CI, −1.099 to −0.0336), with results reaching statistical significance (p = 0.037).

**Table 6:** The Effect of HY and Non-HY Artificial Tear preparations on TBUT: Meta-Analytic Data of RCTs.

Study	No. of subjects in HY arm	No. of subjects in Non-HY arm	Total	SMD	SE	95% CI	t	P	Weight (%)	
									Fixed	Random
Baeyens *et al*.^6^	198	374	572	0.303	0.0882	0.129 to 0.476			51.72	12.65
Baudouin *et al*.^7^	29	37	66	−0.00567	0.245	−0.495 to 0.484			6.70	11.61
Lee *et al*.^9^	64	66	130	−0.283	0.175	−0.630 to 0.0634			13.10	12.17
Benellli *et al*.^10^	20	40	60	−3.652	0.429	−4.512 to −2.793			2.19	9.71
Sanchez *et al*.^11^	15	14	29	−1.657	0.422	−2.522 to −0.792			2.26	9.79
Brignole *et al*.^14^	10	11	21	−0.515	0.427	−1.408 to 0.378			2.21	9.74
Iester *et al*.^18^	58	55	113	0.245	0.188	−0.127 to 0.617			11.44	12.08
Laflamme *et al*.^21^	12	24	36	−0.363	0.348	−1.071 to 0.345			3.32	10.59
Nelson *et al*.^22^	40	30	70	0.0557	0.239	−0.421 to 0.532			7.05	11.66
Total (fixed effects)	446	651	1097	0.0102	0.0635	−0.114 to 0.135	0.161	0.872	100.00	100.00
Total (random effects)	446	651	1097	−0.566	0.271	−1.099 to −0.0336	−2.086	0.037	100.00	100.00
Test for heterogeneity										
Q	14.4555									
DF	6									
Significance level	P = 0.0249									
I^2^ (inconsistency)	58.49%									
95% CI for I^2^	4.19 to 82.02									

HY = Sodium Hyaluronate; SMD = Standard Mean Deviation; SE = Standard Error; CI = Confidence Interval.

**Figure 4:**
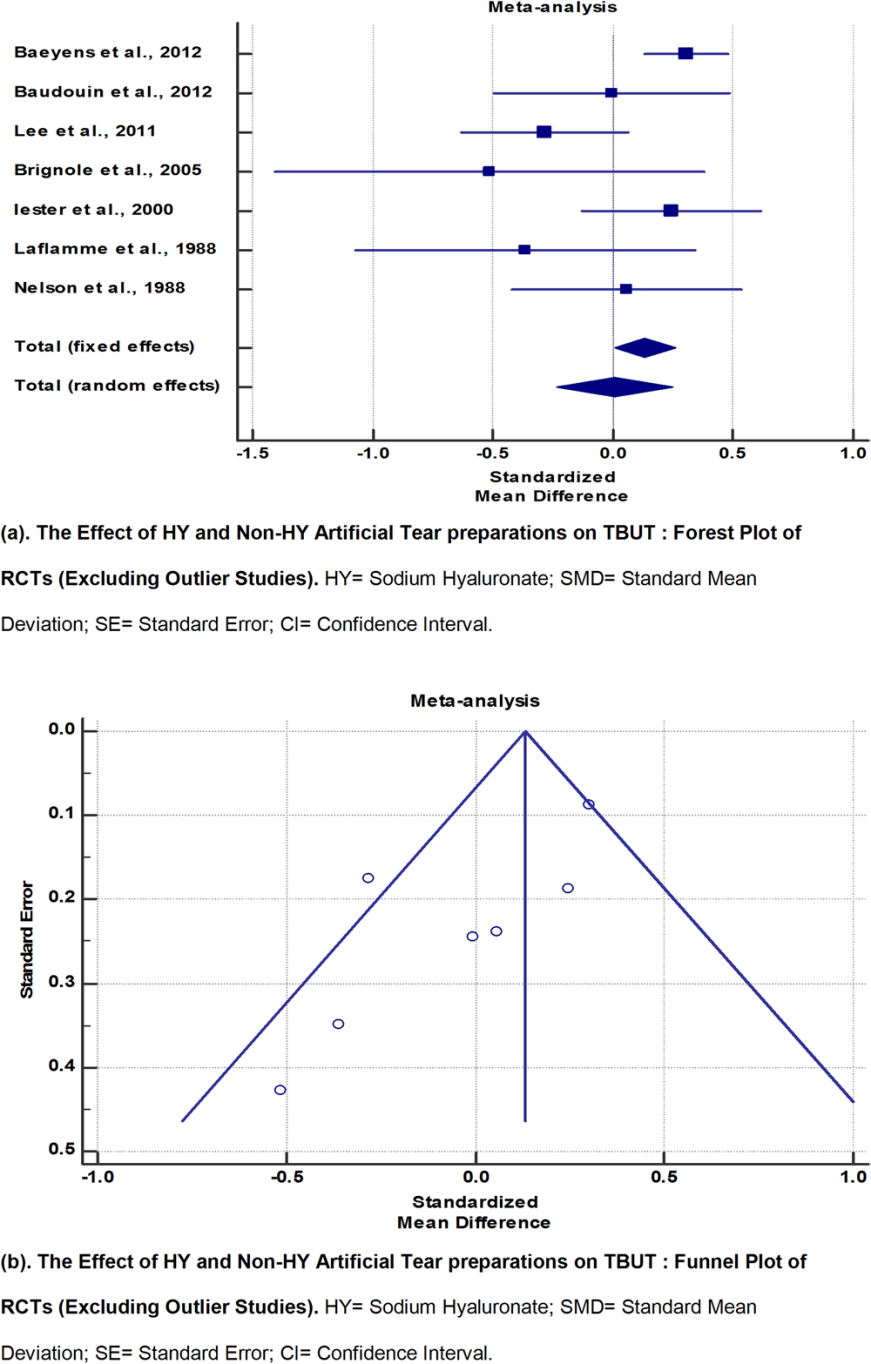
The Effect of HY and Non-HY Artificial Tear preparations on TBUT: Forest Plot and Funnel Plot of RCTs (Excluding Outlier Studies). HY = Sodium Hyaluronate; SMD = Standard Mean Deviation; SE = Standard Error; CI = Confidence Interval.

From the funnel plot, the RCTs by Sanchez *et al*.^11^ and Benelli *et al*.^10^ were identified as outliers. However, upon re-examination of their methodologies, the authors found no justification for exclusion of these studies from the analysis. Nonetheless, a second meta-analysis excluding these 2 studies was run (Fig. 4 and Table 7). While there was greater symmetry of the funnel plot and a lowering of the I^2^ value, the I^2^ value remained above 50%. By random-effects modelling, eyes in the HY group now appeared to demonstrate more improvement of TBUT compared to eyes in the non-HY group (SMD, 0.00761; 95% CI, −0.232 to 0.247). However, the results were not statistically significant (p = 0.95).

**Table 7:** The Effect of HY and Non-HY Artificial Tear preparations on TBUT: Meta-Analytic Data of RCTs (Excluding Outlier Studies).

Study	No. of subjects in HY arm	No. of subjects in Non-HY arm	Total	SMD	SE	95% CI	t	P	Weight (%)	
									Fixed	Random
Baeyens *et al*.^6^	198	374	572	0.303	0.0882	0.129 to 0.476			54.13	24.21
Baudouin *et al*.^7^	29	37	66	−0.00567	0.245	−0.495 to 0.484			7.01	13.10
Lee *et al*.^9^	64	66	130	−0.283	0.175	−0.630 to 0.0634			13.71	17.64
Brignole *et al*.^14^	10	11	21	−0.515	0.427	−1.408 to 0.378			2.31	6.32
Iester *et al*.^18^	58	55	113	0.245	0.188	−0.127 to 0.617			11.97	16.76
Laflamme *et al*.^21^	12	24	36	−0.363	0.348	−1.071 to 0.345			3.47	8.52
Nelson *et al*.^22^	40	30	70	0.0557	0.239	−0.421 to 0.532			7.38	13.45
Total (fixed effects)	411	597	1008	0.134	0.0649	0.00614 to 0.261	2.057	0.040	100.00	100.00
Total (random effects)	411	597	1008	0.00761	0.122	−0.232 to 0.247	0.0623	0.950	100.00	100.00
Test for heterogeneity										
Q	14.4555									
DF	6									
Significance level	P = 0.0249									
I^2^ (inconsistency)	58.49%									
95% CI for I^2^	4.19 to 82.02									

HY = Sodium Hyaluronate; SMD = Standard Mean Deviation; SE = Standard Error; CI = Confidence Interval.

### Qualitative Analyses

Supplementary Tables S1–11 present the extracted data for all outcome measures examined in this analysis. Only outcome measures examined in 2 or more studies are described. Other outcomes examined by only one study and therefore not described in this paper include the tear evaporation rate^8^, tear turnover rate^8^, tear film stability^8^, tear prism height^14^, corneal autofluorescence^16^, tear clearance rate^17^, tear function index^17^, tear ferning pattern test^18^ and corneal sensitivity^20^.

#### Symptoms

15 studies^6–9, 12–16, 18–23^ reported symptoms as an outcome measure, with a total of 20 head-to-head comparison data sets. Majority of the control eyedrops were cellulose-based.

HY demonstrated superiority over control eyedrops in the majority of datasets (10 datasets), with 6 of the 10 demonstrating statistical significance. None of the 4 data sets which demonstrated superiority of the control eyedrop had statistical significance.

Symptoms in DES range from foreign-body sensation, grittiness, red eyes, mucoid discharge, irritation, sensation of dryness and tearing. Symptoms may be multifactorial and influenced directly by the volume (rather than the active component) of eyedrops. Symptoms such as ‘blurring of vision’ may be due also to the viscosity of eyedrops – cellulose and carbomer preprarations may be more viscous than others and cause transient blurring immediately post-instillation. The subjectivity of this outcome measure, wide-range of assessment methods, and inherent inter- and intra-subject variability pose challenges to any systematic review or meta-analysis. This has been attempted previously by Kong X. Y. *et al*.^24^, who were unable to demonstrate unequivocal superiority of HY over other eyedrops in providing subjective relief to DES patients.

#### Ocular Surface Staining

17 studies^6–15, 17–23^ examined ocular staining, with a total of 22 head-to-head comparison datasets. Studies included a range of stains and scoring systems, with examination of either the cornea, conjunctiva or both. If more than one stain was used, fluorescein staining results were extracted. If more than one area was examined, results from corneal staining were extracted.

Majority of the datasets (12 datasets) showed superiority of HY over the control arm, with most (7 datasets) demonstrating statistical significance. Only 5 datasets demonstrated superiority of the control eyedrop and none had statistical significance. The superiority of HY was reported when compared against all 5 different controls – vehicle, PVA, NS, cellulose and carbomer-based eyedrops.

Ocular staining has been used to monitor the severity and progression of DES. Sodium fluorescein permeates intercellular space associated with epithelial cellular disruption; rose bengal stains devitalized cells and mucus through breaks in the tear film; lissamine green stains dead and degenerate cells. The action of HY on CD44 as well as its anti-inflammatory effects may therefore assume greater significance here, having been shown to improve corneal epithelial repair, cellular migration and stabilization of the ocular barrier.

Unfortunately, analyses of this outcome measure is limited by the lack of standardized methodologies and stains. In addition, while some used named grading systems such as the Oxford Scheme and Van Bijsterveld Scoring System, the majority did not specify any grading system. Given this heterogeneity, we attempted to limit our data extraction to results reflecting only cornea staining (with or without the conjunctiva) with fluorescein.

#### Non-Invasive Tear Breakup Time (NITBUT)

2 studies^8, 13^ examined NIBUT, comparing 0.1–0.15% HY to CMC and carbomer. The 2 studies utilized the Hir-Cal Grid with a keratometer^8^ or the Tearscope Plus (Keeler Limited, Windsor, UK). Only Johnson *et al*.^13^ reported their results and did not find a statistically nor clinically significant difference between the effects of both arms on NITBUT.

#### Tear Osmolarity

5 studies^7, 8, 18, 22^ examined tear osmolarity, with a total of 6 head-to-head comparison datasets. Majority of the control eyedrops were cellulose-based. HY was shown to be superior in 3 of the 5 datasets, with 1 dataset demonstrating statistical significance.

Studies exhibited a range of results. Benelli *et al*.^10^ demonstrated improvement in all arms within the first 30 days, while showing superiority of HY. Results across day 15 to day 60 from Iester *et al*.^18^ similarly favoured HY. The remaining studies did not show any difference in effect between groups across 30 days to 3 months.

Studies used different measurement techniques and varying concentrations of eyedrops. Studies which demonstrated superiority of HY appeared to use higher concentrations (0.2 to 0.4%) compared to the other studies (0.1 to 0.18%). The varying hypotonicity of eyedrops may also influence the tear osmolarity results^25^.

Tear osmolarity is a worthwhile outcome measure in any DES study, having been suggested to be the most objective test for DES^26^. While an unclear cut-off value for the diagnosis of DES has previously limited the usefulness of this test^26^, the diagnostic threshold of ≥316 mOsm/L has now been validated^27^. While previous assessment methods were laboratory-based and time-consuming, recent ones are easy-to-use and require minimal tear sample, as that utilized by Benelli *et al*.^10^. Finally, from the pathophysiological perspective, tear osmolarity indirectly assesses the rate of tear secretion and loss by evaporation^28^ resulting from an unstable tear film or reduced volume, therefore representing a meaningful indicator of DES.

#### Tear Meniscus

3 studies^17, 21, 23^ examined tear meniscus as an outcome measure, using 0.1% HY as the study eyedrop and 1–1.4% PVA as controls. Laflamme *et al*.^21^ used an arbitrary scale based on assessment of the meniscus as ‘normal’ or ‘absent’ and showed superiority of 1.4% PVA, but with only a small sample size not achieving statistical significance. MacDonald *et al*.^16^ reported that the tear menisci of subjects in both arms remained unchanged throughout the study.

#### Conjunctival Impression

5 studies^11, 14, 15, 18, 22^ examined conjunctival impression – with 2 studies^11, 14^ utilising flow cytometry and the other 3 studies^15, 18, 22^ grading the appearance of epithelial and goblet cells.

Sanchez *et al*.^11^ and Brignole *et al*.^14^ utilised flow cytometry and demonstrated underexpression of HLA-DR in both the HY and control arms, with Sanchez *et al*. demonstrating greater underexpression in the carmellose arm. This study also showed, without statistical significance, a trend toward decreased expression of other inflammatory markers, including CD11b and CD3. Brignole *et al*.^14^ took its first measurements at day 56 and demonstrated a greater decrease in CD44 in the HY group compared to the carboxymethylcellulose (CMC) group. This study further assessed markers understood to be protective in nature – MUC5AC, CD63 and UIC2 – and found these to be increased in both groups, although without statistical significance.

Aragona *et al*.^15^, Iester *et al*.^18^ and Nelson *et al*.^22^ utilized impression cytology scores to reflect qualitative analysis of epithelial and goblet cells. Aragona *et al*. and Nelson *et al*. graded specimens utilizing a common method^29^, while Iester *et al*. used a different 3-point average scoring system.

Aragona *et al*.^15^ demonstrated statistically significant results only after 3 months of treatment, with saline-treated eyes having higher cytology scores (less keratinised basophilic cytoplasm, greater nucleus/cytoplasm ratio and increased goblet cells) compared to the HY arm. This was postulated to be due to the detrimental long-term effect of saline on the ocular surface. Iester *et al*.^18^ showed a statistically significant improvement in impression cytology in the HY arm compared to the hydroxypropylmethylcellulose arm at day 90. Nelson *et al*.^22^ could not demonstrate any inter-group differences, however showed a statistically significant improvement from baseline in the HY group at 8 weeks.

The late effects of the eyedrops on conjunctival impression cytology and flow cytometry are unsurprising given the long-term nature of cellular turnover and molecular production. This lies beyond the scope of this study, which extracted data up to only 5 weeks after initiation of eyedrops.

#### Corneal Topography

2 studies^10, 14^ utilized corneal topography – Benelli *et al*.^10^ measured the effect of different eyedrops on wavefront aberrometry while Brignole *et al*.^14^ measured corneal surface regularity index (SRI). Brignole *et al*. demonstrated better SRI outcomes for the HY group, suggesting the presence of greater uniformity in ocular surface protection by HY compared to CMC. Benelli *et al*.^10^ showed better outcomes for CMC in wavefront aberrometry, however results were not significant.

#### Visual Acuity

4 studies^6, 10, 22, 23^ examined visual acuity (VA) as an outcome measure, with a total of 6 head-to-head comparison datasets. Majority of the studies reported neither their results nor methodologies in detail. Only Benelli *et al*.^10^ described VA testing under controlled, standardized environments and showed that HY was superior over CMC and HP Guar, However, this was not statistically significant.

VA is a useful patient-centered outcome and improvement may result from a better tear film quality and ocular surface. However, VA is unavoidably affected by numerous other ocular factors, presenting an inherent limitation in its usefulness as an efficacy measure for ocular surface treatment.

## Discussion

The studies reviewed in this paper demonstrated an improvement from baseline across most of the outcome measures for both HY and non-HY based lubricants. However, neither qualitative nor quantitative analyses could reveal consistent nor clinically significant superiority of one treatment arm over the other.

Meta-analyses for SH I and TBUT data revealed that while HY-based preparations appeared superior in improving SH I, non-HY based preparations were more effective in improving TBUT.

However, it should be noted that the SMDs between preparations were small - less than 1mm for SH I (0.238mm) and less than 1 second for TBUT (0.566 seconds). These differences, while statistically significant, are unlikely to have clinical significance – firstly, poor repeatability has been demonstrated for measurements of both SH I and TBUT under various conditions^24, 30^ and between measuring techniques^24^. A difference of 3 seconds may be expected between any two separate measurements of TBUT^24^, suggesting that any difference of less than 3 seconds may not be clinically significant. Secondly, some have suggested that the variability in the SH I test (without anesthesia) is likely to be greater than the SH II (with anesthesia) test as SH I is influenced by reflex, in addition to baseline tear secretion^31^. Finally, these small differences are unlikely to have significant effect on patient symptoms, given the numerous inherent and environmental factors which have been shown to influence TBUT and SH I measurements^32^.

As such, the small magnitude of difference for both TBUT and SH I found in the studies we analysed is likely to be well within the expected variability for both outcome measures.

This may reflect true comparability between HY and non-HY preparations. Individually, both HY and non-HY preparations have been demonstrated in most studies to be effective. Their respective mechanisms of action appear to overlap, and it is unsurprising that both would exhibit efficacy in improving both TBUT and SH I, given the basis of these tests.

The effect of HY in the treatment of DES is likely the result of various mechanisms of action. Firstly, *in*-*vitro* studies^29^ have demonstrated that HY inactivates the CD44 adhesion molecule, a receptor of HY found to be over expressed in cornea and conjunctiva cells of subjects with DES. This HY-CD44 interaction has been shown to stabilise the ocular surface barrier and tear film^33^, creating a favourable ocular surface microenvironment which increases cell adhesion and motility^34^ and promotes cellular migration^35^. Secondly, HY has been postulated to have localised anti-inflammatory effects – particularly in patients with at least moderate dry eyes and superficial keratitis^36^. Thirdly, the high viscosity of HY reduces friction between the cornea and eyelids during extraocular movements and blinking, therefore reducing mechanical damage of the cornea^33^. Fourthly, HY has significant water-retentive properties – with an affinity of 1000-fold its own weight. This increases ocular surface wettability^37^ and reduces tear evaporation^38^.

It is important to realize that many of the comparison groups in published studies are not entirely passive controls but do share some mechanisms of action with HY, which may conceivably improve SH I and TBUT. Carbomer, methylcellulose and PVA are all viscosity-enhancing agents used in eyedrop preparations. Methylcellulose is viscous, anionic and possesses significant bioadhesive characteristics^39, 40^ which increase the tear retention time^41^. PVA has film-forming, emulsifying, adhesive properties^42^ and at the same viscosity, produces a thicker film than other polymers^43^.

The insignificant difference between HY and non-HY preparations may also be related to the inherent lack of precision and specificity of the SH and TBUT tests. This may also explain the apparent efficacy of placebo and vehicle preparations.

SH I (without anesthesia) reflects both reflex and basal aqueous tear production and remains a common test for diagnosis and monitoring of DES. Unfortunately, this test possesses numerous limitations. These include low reproducibility, low sensitivity and specificity, lack of a standardized site of paper strip placement, uneven absorption of tears by the strip, uncertainty regarding the relationship between the quantity of fluid absorbed by strips with its wetted length and lack of a standardized method of evaluation of the wetted length^44^. Fujihara T *et al*.^45^ concluded that SH I lacks sufficient sensitivity to be a meaningful test for patients with DES that is less than severe. SH I results may improve even with inactive ‘placebo’ preparations, due to their direct effect in increasing tear volume, reducing osmotic pressure, reducing ocular surface friction, and in diluting pro-inflammatory tear substances.

Unlike SH I, TBUT primarily assesses tear film instability in evaporative dry eye disease^41^. However, again the ‘break up’ of the film may occur due to various mechanisms, including the diffusion and absorption of the lipid layer into the mucous-aqueous interface^46^ or the rupture of the mucous layer at its weakest spots. Like SH I, TBUT has been labelled inaccurate and non-reproducible^47^, with lack of a standardized procedure for fluorescein application. It may be poorly associated with subjective dry eye symptoms^48^.

Qualitative analyses suggest the superiority of HY over the respective control arms in the assessment of SH I, ocular staining, symptoms and tear osmolarity. Neither appeared to be superior in conjunctival impression results. This study did not attempt to draw conclusions from the observation of visual acuity, tear meniscus, NITBUT and corneal topography results, as these outcomes were reported in less than 4 studies each.

The main limitation of our study pertains to the heterogeneity of included studies. Studies differed in study design (parallel vs. crossover), concentration of HY and control eyedrops, eyedrop application regime, follow up duration, baseline characteristics of subjects (differences in age, gender and severity of DES), definition of DES itself^49, 50^, control eyedrop, as well as the presence of preservatives in the preparations. All these factors may influence the results of this review.

This study has attempted to mitigate this heterogeneity, to enhance the meaningfulness and applicability of the results in this review. Firstly, key characteristics for each study are detailed in Table 2 to allow better contextualization of our results. Secondly, all studies used a HY concentration of at least 0.1%, previously demonstrated as the minimum concentration to have an effect on patients with DES^51^. Thirdly, this review has attempted to ensure that data extracted across all studies is as homogenous as possible. For example, data has been restricted to that obtained at the earliest time point after the first week, but no later than 35 days after eyedrop instillation. In addition, only studies deemed to have utilized a ‘non-active’ compound in their control arm have been included.

The second limitation of our study pertains to the nature of tests for DES. The lack of a standardized protocol for DES tests has resulted in studies differing on methodologies as well as the quantification of results for the same outcome measure. The limitations of SH I and TBUT have been described above. These factors may confound the results of a meta-analysis which assumes homogeneity in the methodology of outcome measures across various studies^52^.

Thirdly, most of the studies did not compare HY against its pure vehicle. The differences in molecular weight, tonicity, preservatives and mineral composition between HY and non-HY eyedrops may further influence results^53, 54^.

Finally, in the interest of reducing heterogeneity among selected studies, data from a relatively narrow window post-initiation of eyedrops was included for analysis. However, depending on the outcome measure, the effect of different lubricant preparations may be observed at other time points. For example, owing to its unique tear stabilizing properties, HY may reduce symptoms and alleviate keratitis within a shorter time than other preparations^13, 52^. Differences in effect on conjunctival impression and cytology are likely to manifest only later. Therefore, it should be emphasized that this review compares only the short-term/early efficacies of the various artificial tear preparations.

## Conclusion

This review examined 18 clinical trials comparing the efficacy of HY against alternative lubricant preparations. Superiority of one preparation over another could not be consistently demonstrated across the range of outcome measures. While meta-analyses demonstrate that HY may provide superior benefit in SH I and non-HY preparations may be superior in improving the TBUT, results were not clinically significant. Overall, this may reflect true comparability in efficacy between HY-based and non-HY preparations. Heterogeneity across studies presents the main limitation to this study, suggesting the need for a large RCT with standardized protocols to properly evaluate the comparative efficacy of artificial tear preparations.

## Methods

### Literature Search Strategy

An electronic literature search was carried out through the PubMed, Cochrane Central Register of Controlled Trials and Scopus databases from inception up to May 2016. Databases were searched using the terms (“hyaluronic acid” OR “hyaluronate”) AND (“dry eye” OR “keratoconjunctivitis sicca”). Limits were applied to select for only clinical trials performed on human subjects.

### Inclusion Criteria

Only *in*-*vivo*, prospective, interventional trials examining the effect of HY on the ocular surface were considered for inclusion. Further, studies were included if they: (1) employed at least one HY-only arm; (2) employed at least one non-HY arm, which was determined to use a ‘non-active’ compound (see Table 1) –labelled the ‘control’ arm; (3) had a follow-up period of at least one week after initiation of eyedrops; (4) excluded subjects who were on long-term eyedrops (e.g. glaucoma medications) or contact lens wear; (5) did not perform intra-subject comparison (i.e. comparison of the effect of an eyedrop in one eye and a different eyedrop in the other); (6) were published in English; (7) were not conducted on post-refractive surgery patients.

Both parallel and crossover studies were included in this study.

### Study Selection

The abstracts of all studies were read by at least 2 of the authors (BA, JS or PW). Where there were no obvious reasons for immediate exclusion, the full article was reviewed by at least 2 of the above authors. The eligibility of each study was determined unanimously, with strict guidance from the predetermined inclusion criteria. Any discordance regarding a study’s suitability for inclusion was resolved through discussion with the senior author (LT).

### Data Extraction

Relevant information and data from the studies were extracted and entered into 3 databases.

The first database included the name of the first author, year of publication, country of study and other study characteristics such as randomization, masking, total duration of follow-up, mean/median age of subjects, severity of DES, % preparation of HY and control eyedrop used (Table 2).The outcome measures obtained in each study were summarized (Table 3).

The second database included results of the outcome measures from the studies, including the number of subjects analysed for each outcome measure, the arm which demonstrated greater comparative efficacy as well as the difference in treatment effect and p-value, if provided in the study. In crossover trials with more than one test period of the same eyedrop medication, only the results of the first trial were included for analysis (unless the results were that from analysis which had already included the consequent test periods).

For studies which had multiple data/outcome measurement points, only the earliest measurement taken between 1 to 5 weeks post-commencement of eye drops was used for analysis. Any measurements taken after 5 weeks post-commencement of eye drops were excluded from data extraction and analysis. Studies with multiple control eyedrop arms or separate results for right and left eyes of subjects have their results presented as separate data entries, hereafter referred to as ‘datasets’. Only results of outcome measures which were examined in 2 or more studies are presented (Supplementary Tables S1–11). This is with the exception of the outcome measure of “safety”. Although “safety” was examined in 8 studies, this outcome measure was deemed too heterogenous, with too large a range of specific “safety” parameters for meaningful comparison and analysis.

The final database extracted the quantitative results reported from randomized controlled trials (RCTs) which studied SH I and/or TBUT. Further meta-analysis was conducted for studies which reported both baseline and follow-up values and/or the change from baseline to follow-up values, for both the HY and non-HY arms. Corresponding authors of studies which did not report the complete set of data as above were contacted via their correspondence email addresses, in an attempt to obtain the missing data. For crossover studies^19, 21^, the authors agreed that only data from the *first* trial and the right eye (where applicable)^19^ was included for analysis. For studies with more than one non-HY intervention group^6, 7^, 3 in order to avoid unit-of-analysis error, non-HY groups were combined into a single group and compared against the HY treatment arm.

### Statistical Analysis

Meta-analyses for both SH I and TBUT as outcome measures were performed with MedCalc for Windows, Version 16.8 (MedCalc Software, Ostend, Belgium). Where the standard deviation of change was not reported in the study, it was imputed with guidance from the *Cochrane Handbook for Systematic Reviews of Interventions* ^55^, with Corr, the imputed correlation coefficient, taken as 0.5 in this study.

Standardized mean difference (SMD) was used for the continuous data and 95% confidence intervals (CIs) were calculated for summary estimates. A p-value of less than 0.05 was considered statistically significant. The χ^2^-basedI^2^ statistic (expressed as a percentage) was used to measure heterogeneity among the trials. An I^2^ value of more than 50% was considered suggestive of significant statistical heterogeneity. Both fixed and random-effects modelling were applied in analyses.

### Risk of Bias Assessment

All the studies included for meta-analysis were assessed for risk of bias using the Cochrane Risk of Bias tool^56^. This tool evaluates studies for risk of selection bias, performance bias, attrition bias, reporting bias and other types of bias. 2 authors (BA and JS) independently assessed the risk of bias. Discrepancies were resolved by discussion and consultation with the senior author (LT)^57^.

Funnel plots of the intervention effect estimates were developed and examined for evidence of asymmetry. A symmetric funnel plot reflects less bias while an asymmetric funnel plot may imply possible selection/publication bias, poor reporting of small trials, chance or true heterogeneity.

## Electronic supplementary material

Supplementary Information 1
