# Supplementary material for: Sodium Hyaluronate in the Treatment of Dry Eye Syndrome: A Systematic Review and Meta-Analysis
Source: Sci Rep. 2017 Aug 21;7:9013. doi: 10.1038/s41598-017-08534-5 (PMC5567178; doi:10.1038/s41598-017-08534-5)
Supplement: Supplementary file 1 — Supplementary Information 1 [file 41598_2017_8534_MOESM1_ESM.pdf]

Sodium Hyaluronate in the Treatment of Dry Eye Syndrome: A Systematic Review and Meta-Analysis

ANG Bryan Chin Hou[1,2,], SNG James Jie[1,**], WANG Priscilla Xin Hui[1], HTOON Hla Myint[3] and, TONG Louis Hak Tien[2,3,*].**

* Corresponding Author

** Joint First Authors

[1] National Healthcare Group Eye Institute, Tan Tock Seng Hospital, Singapore

[2] Singapore National Eye Centre, Singapore

[3] Singapore Eye Research Institute, Singapore

Corresponding Author:

TONG Louis Hak Tien

Singapore Eye Research Institute, Singapore

The Academia, 20 College Road

Discovery Tower Level 6

Singapore 169856

Telephone: 65-6322 4500 / Fax: 65-6225 2568

E-mail: louis.tong.h.t@singhealth.com.sg

Conflict of Interest / Financial Disclosure(s):

Nil

Key Words:

Sodium Hyaluronate, Hyaluronic Acid, Dry Eye Syndrome, Xerophthalmia, Systematic Review, Meta-Analysis

Tear Breakup Time						
Study (Year)	Study Arms	Number of Patients Analyzed for Outcome Measure (Treatment, Control)	Superior Eyedrop	P value	Difference in Treatment Effect (HY vs. Control) / Effect Magnitude (HY vs. Control) / Test for Treatment Effect (95% CI)	Remarks
Baeyens (2012)	0.18% HY; Normal Saline	195 (99, 96)	HY	0.1182	1.5 ± 3.84 vs. 0.73 ± 2.06	
Baeyens (2012)	0.18% HY; 0.3% Carbomer	190 (99, 91)	HY	N.A	1.5 ± 3.84 vs. 0.59 ± 1.88	
Baudouin (2012)	0.18% HY; 0.5% CMC	66 (29, 37)	CMC	N.A	0.04 (-0.78, 0.86)	
Lee (2011)	0.1% HY; 0.5% CMC	65 (32, 33)	CMC	>0.05	1.10 ± 1.83 vs. 1.50 ± 1.68	
Benelli (2010)	0.2% HY; 0.5% CMC	40 (20.20)	CMC	1.0	-13.3 (6.8) vs. 1.3 (1.1)	
Benelli (2010)	0.2% HY; 0.18% HP Guar	40 (20.20)	HP Guar	0.5635	-13.3 (6.8) vs. 1.6 (1.0)	
Sanchez (2010)	0.15% HY; 0.5% Carmellose	15 (8, 7)	Carmellose	N.A	-0.1 vs. 2.3	
Johnson (2008)	0.1% HY; 0.3% Carbomer	65 (32, 33)	N.A	0.35	(-0.38, 1.04)	Data reflects Carbomer-HY results
Brignole	0.18% HY;	21 (10, 11)	NIL	>0.05	1 vs. 1	

Tear Breakup Time						
Study (Year)	Study Arms	Number of Patients Analyzed for Outcome Measure (Treatment, Control)	Superior Eyedrop	P value	Difference in Treatment Effect (HY vs. Control) / Effect Magnitude (HY vs. Control) / Test for Treatment Effect (95% CI)	Remarks
(2005)	1% CMC					
Aragona (2002)	0.15% HY; 0.9% Saline	44 (19, 25)	NIL	N.S	N.A	
MacDonald (2002)	0.1% HY; 1.4% PVA	32	N.A	0.4	-2.0, 0.3	Right Eye
MacDonald (2002)	0.1% HY; 1.4% PVA	32	N.A	0.16	-1.9, 0.8	Left Eye
Iester (2000)	0.4% HY; 0.3% CMC	113 (58, 55)	HY	<0.01	1.73 vs. 1.02	
Sand (1989)	0.1% HY; Placebo	18	NIL	N.A	N.A	
Sand (1989)	0.2% HY; Placebo	18	HY	<0.005	N.A	
Laflamme (1988)	0.1% HY; 1.4% PVA	12	1.4% PVA	N.A	-0.29 vs 1.17	
Nelson (1988)	0.1% HY; 1.4% PVA	35 (20, 15)	HY	N.S	1.2 vs. 1	
Limberg (1987)	0.1% HY; 1% PVA	20	N.A	N.A	N.A	

Supplementary Table S1. Summary of Results of Studies, by Outcome Measure (TBUT). TBUT= Tear Breakup Time; HY= Hyaluronic Acid; PVA= Polyvinyl Alcohol; CMC= Carboxymethylcellulose.

SH I						
Study (Year)	Study Arms	Number of Patients Analyzed for Outcome Measure (Treatment, Control)	Superior Eyedrop	P value	Difference in Treatment Effect (HY vs. Control) / Effect Magnitude (HY vs. Control) / Test for Treatment Effect (95% CI)	Remarks
Baeyens (2012)	0.18% HY; Normal Saline	185 (89, 96)	HY	0.0598	2.26 ± 5.58 vs. 1.31 ± 4.35	
Baeyens (2012)	0.18% HY; 0.3% Carbomer	180 (89, 91)	HY	N.A	2.26 ± 5.58 vs. 1.52 ± 3.80	
Baudouin (2012)	0.18% HY; 0.5% CMC	66 (29, 37)	HY	N.A	-1.1 (-3.6, 1.4)	
Benelli (2010)	0.2% HY; 0.5% CMC	40 (20.20)	HY	0.0537	2.5 (1.7) vs. 0.9 (2.8)	
Benelli (2010)	0.2% HY; 0.18% HP Guar	40 (20.20)	HY	0.0178	2.5 (1.7) vs. 0.6 (1.8)	
Vogel (2010)	0.18% HY; Vehicle	436 (217, 219)	NIL	N.S	N.A	
MacDonald (2002)	0.1% HY; 1.4% PVA	32	N.A	1.00	-1.3, 1.3	Right Eye
MacDonald (2002)	0.1% HY; 1.4% PVA	32	N.A	0.43	-2.2, 1.0	Left Eye
Iester (2000)	0.4% HY; 0.3% CMC	113 (58, 55)	HY	<0.0001	3.19 vs 0.87	

SH I						
Study (Year)	Study Arms	Number of Patients Analyzed for Outcome Measure (Treatment, Control)	Superior Eyedrop	P value	Difference in Treatment Effect (HY vs. Control) / Effect Magnitude (HY vs. Control) / Test for Treatment Effect (95% CI)	Remarks
Condon (1999)	0.1% HY; 0.9% Saline	70	HY	0.0006	3.6	
Sand (1989)	0.1% HY; Placebo	18	NIL	N.A	N.A	
Sand (1989)	0.2% HY; Placebo	18	NIL	N.A	N.A	
Laflamme (1988)	0.1% HY; 1.4% PVA	12	1.4% PVA	N.A	0.46 vs. 2.33	
Nelson (1988)	0.1% HY; 1.4% PVA	35 (20, 15)	1.4% PVA	N.S	1.8 vs. 2.2	
Limberg (1987)	0.1% HY; 1% PVA	20	N.A	N.A	N.A	

Supplementary Table S2. Summary of Results of Studies, by Outcome Measure (SH I). SH I=Schirmer's Test I (without anaesthesia); HY= Hyaluronic Acid; PVA= Polyvinyl Alcohol; CMC= Carboxymethylcellulose.

Ocular Staining						
Study (Year)	Study Arms	Number of Patients Analyzed for Outcome Measure (Treatment, Control)	Superior Eyedrop	P value	Difference in Treatment Effect (HY vs. Control) / Effect Magnitude (HY vs. Control) / Test for Treatment Effect (95% CI)	Remarks
Baeyens (2012)	0.18% HY; Normal Saline	195 (99, 96)	HY	0.0074	-1.42 ± 1.45 vs. -0.97 ± 1.27	Fluorescein
Baeyens (2012)	0.18% HY; 0.3% Carbomer	190 (99, 91)	HY	N.A	-1.42 ± 1.45 vs. -1.04 ± 1.23	Fluorescein
Baudouin (2012)	0.18% HY; 0.5% CMC	66 (29, 37)	CMC	N.A	-0.3 (-1.1, 0.6)	Cornea and conjunctiva
McCann (2012)	0.15% HY; CMC	49 (24,25)	N.A	N.A	N.A	Cornea fluorescein
Lee (2011)	0.1% HY; 0.5% CMC	65 (32, 33)	CMC	>0.05	-2.00 ± 1.27 vs. -2.60 ± 1.41	Cornea
Benelli (2010)	0.2% HY; 0.5% CMC	40 (20.20)	HY	0.2733	35% vs. 15% improved 1 grade	Cornea and conjunctiva fluorescein
Benelli (2010)	0.2% HY; 0.18% HP Guar	40 (20.20)	HP Guar	0.7475	35% vs. 45% improved 1 grade	Cornea and conjunctiva fluorescein
Sanchez (2010)	0.15% HY; 0.5% Carmellose	15 (8, 7)	Carmellose	N.A	0 vs. 1	Cornea fluorescein
Vogel	0.18% HY;	436 (217, 219)	HY	0.024	-0.4	Cornea and conjuctiva lissamine

Ocular Staining						
Study (Year)	Study Arms	Number of Patients Analyzed for Outcome Measure (Treatment, Control)	Superior Eyedrop	P value	Difference in Treatment Effect (HY vs. Control) / Effect Magnitude (HY vs. Control) / Test for Treatment Effect (95% CI)	Remarks
(2010)	Vehicle					green
Vogel (2010)	0.18% HY; Vehicle	436 (217, 219)	NIL	N.S	N.A	Cornea fluorescein
Johnson (2008)	0.1% HY; 0.3% Carbomer	65 (32, 33)	HY	0.036	0.22 log units	Cornea fluorescein
Brignole (2005)	0.18% HY; 1% CMC	21 (10, 11)	HY	>0.05	-2.7 vs. -2.3	Cornea fluorescein
Aragona (2002)	0.15% HY; 0.9% Saline	86 (41, 45)	NIL	N.S	N.A	Fluorescein and rose bengal
MacDonald (2002)	0.1% HY; 1.4% PVA	32	HY	0.04	-1.6, -0.05	Right eye. Cornea and conjunctiva rose bengal
MacDonald (2002)	0.1% HY; 1.4% PVA	32	HY	0.37	-1.0, 0.4	Left eye. Cornea and conjunctiva rose bengal
Iester (2000)	0.4% HY; 0.3% CMC	113 (58, 55)	HY	<0.0001	-1.61 vs. -1.03	"Ocular surface" fluorescein
Condon (1999)	0.1% HY; 0.9% Saline	70	HY	0.0001	N.A	Cornea and conjunctiva rose bengal
Sand (1989)	0.1% HY; Placebo	18	NIL	N.A	N.A	Rose bengal

Ocular Staining						
Study (Year)	Study Arms	Number of Patients Analyzed for Outcome Measure (Treatment, Control)	Superior Eyedrop	P value	Difference in Treatment Effect (HY vs. Control) / Effect Magnitude (HY vs. Control) / Test for Treatment Effect (95% CI)	Remarks
Sand (1989)	0.2% HY; Placebo	18	HY	<0.005	N.A	Rose bengal
Laflamme (1988)	0.1% HY; 1.4% PVA	12	1.4% PVA	NIL	-0.41 vs. -0.48	Cornea
Nelson (1988)	0.1% HY; 1.4% PVA	35 (20, 15)	HY	N.S	-0.6 vs. -0.2	Cornea and conjunctiva rose bengal
Limberg (1987)	0.1% HY; 1% PVA	20	N.A	N.A	N.A	

Supplementary Table S3. Summary of Results of Studies, by Outcome Measure (Ocular Staining). HY= Hyaluronic Acid; PVA= Polyvinyl Alcohol; CMC= Carboxymethylcellulose.

Symptoms						
Study (Year)	Study Arms	Number of Patients Analyzed for Outcome Measure (Treatment, Control)	Superior Eyedrop	P value	Difference in Treatment Effect (HY vs. Control) / Effect Magnitude (HY vs. Control) / Test for Treatment Effect (95% CI)	Remarks
Baeyens (2012)	0.18% HY; Normal Saline	195 (99, 96)	HY	0.1738	-61.75 ± 78.85 vs. -55.03 ± 83.35	Symptom Intensity
Baeyens (2012)	0.18% HY; 0.3% Carbomer	190 (99, 91)	Carbomer	N.A	-61.75 ± 78.85 vs. -65.85 ± 68.07	Symptom Intensity
Baudouin (2012)	0.18% HY; 0.5% CMC	64 (27, 37)	CMC	0.257	-4.08 (-11.23 to 3.07)	ODSI score
McCann (2012)	0.15% HY; CMC	49 (24,25)	N.A	N.A	N.A	
Lee (2011)	0.1% HY; 0.5% CMC	65 (32, 33)	HY	>0.05	-5.40 ± 5.49 vs. -4.40 ± 4.34	
Vogel (2010)	0.18% HY; Vehicle	436 (217, 219)	NIL	N.S	N.A	VAS for symptom intensity, symptom frequency
Johnson (2008)	0.1% HY; 0.3% Carbomer	65 (32, 33)	NIL	0.94	(-2.6, 2.4 units)	OCI
Brignole (2005)	0.18% HY; 1% CMC	21 (10, 11)	HY	0.039	80% vs. 30%	Subjective comfort score: % of subjects rating comfort as 'good'
Aragona (2002)	0.15% HY; 0.9% Saline	86 (41, 45)	NIL	N.S	N.A	
MacDonald (2002)	0.1% HY; 1.4% PVA	32	HY	0.03	-12.3 (-23.5 to -1.1)	VAS score for 'burning sensation'
Iester (2000)	0.4% HY; 0.3% CMC	113 (58, 55)	HY	<0.05	N.A	Photohobia, pain

Symptoms						
Study (Year)	Study Arms	Number of Patients Analyzed for Outcome Measure (Treatment, Control)	Superior Eyedrop	P value	Difference in Treatment Effect (HY vs. Control) / Effect Magnitude (HY vs. Control) / Test for Treatment Effect (95% CI)	Remarks
Iester (2000)	0.4% HY; 0.3% CMC	113 (58, 55)	HY	<0.0001	N.A	Burning, Foreign body sensation
Condon (1999)	0.1% HY; 0.9% Saline	70	HY	<0.01	N.A	Duration of relief of 'burning sensation'
Condon (1999)	0.1% HY; 0.9% Saline	70	HY	0.002	N.A	Duration of relief of 'grittiness'
Sand (1989)	0.1% HY; Placebo	18	NIL	N.A	NIL	VAS
Sand (1989)	0.2% HY; Placebo	18	NIL	N.A	NIL	VAS
Laflamme (1988)	0.1% HY; 1.4% PVA	10	HY	N.A	-2.8 vs. -1.3	VAS for 'burning and irritation'
Laflamme (1988)	0.1% HY; 1.4% PVA	10	HY	N.A	-1.75 vs. -0.5	VAS for 'foreign body sensation'
Nelson (1988)	0.1% HY; 1.4% PVA	35 (20, 15)	1.4% PVA	N.S	-21.8 vs. -22.7	VAS for 'pain/discomfort'
Limberg (1987)	0.1% HY; 1% PVA	20	1% PVA	N.A	N.A	

Supplementary Table S4. Summary of Results of Studies, by Outcome Measure (Symptoms). HY= Hyaluronic Acid; PVA= Polyvinyl Alcohol; CMC= Carboxymethylcellulose; ODSI= Ocular Surface Disease Index; VAS= Visual Analogue Scale; OCI= Ocular Comfort Index.

Tear Osmolality						
Study (Year)	Study Arms	Number of Patients Analyzed for Outcome Measure (Treatment, Control)	Superior Eyedrop	P value	Difference in Treatment Effect (HY vs. Control) / Effect Magnitude (HY vs. Control) / Test for Treatment Effect (95% CI)	Remarks
Baudouin (2012)	0.18% HY; 0.5% CMC	23 (12, 11)	CMC	N.A	-0.8 (-17.2, 15.6)	
McCann (2012)	0.15% HY; CMC	49 (24,25)	Nil	N.S	N.A	
Benelli (2010)	0.2% HY; 0.5% CMC	40 (20.20)	HY	0.1704	-4.1 (2.9) vs -2.6 (2.4)	
Benelli (2010)	0.2% HY; 0.18% HP Guar	40 (20.20)	HY	0.6926	-4.1 (2.9) vs -4.8 (2.9)	
Iester (2000)	0.4% HY; 0.3% CMC	57	HY	<0.001	-48 vs. -10	
Nelson (1988)	0.1% HY; 1.4% PVA	35 (20, 15)	1.4% PVA	N.S	-25.8 vs. -58.3	

Supplementary Table S5. Summary of Results of Studies, by Outcome Measure (Tear Osmolality). HY= Hyaluronic Acid; PVA= Polyvinyl Alcohol; CMC= Carboxymethylcellulose.

Conjunctival Impression with Flow Cytometry						
Study (Year)	Study Arms	Number of Patients Analyzed for Outcome Measure (Treatment, Control)	Superior Eyedrop	P value	Difference in Treatment Effect (HY vs. Control) / Effect Magnitude (HY vs. Control) / Test for Treatment Effect (95% CI)	Remarks
Sanchez (2010)	0.15% HY; 0.5% Carmellose	15 (8, 7)	Carmellose	<0.05	36.7 ± 29.3 (20.4, 53) vs. 8.9 ± 9.9 (3.2, 14.7)	HLA-DR results presented (as % of positive cells). No significant difference for CD11b and CD3.
Brignole (2005)	0.18% HY; 1% CMC	21 (10, 11)	NIL	N.A	N.A	

Supplementary Table S6. Summary of Results of Studies, by Outcome Measure (Conjunctival Impression with Flow Cytometry). HY= Hyaluronic Acid; CMC= Carboxymethylcellulose.

Conjunctival Impression with Cytology						
Study (Year)	Study Arms	Number of Patients Analyzed for Outcome Measure (Treatment, Control)	Superior Eyedrop	P value	Difference in Treatment Effect (HY vs. Control) / Effect Magnitude (HY vs. Control) / Test for Treatment Effect (95% CI)	Remarks
Aragona (2002)	0.15% HY; 0.9% Saline	86 (41, 45)	NIL	N.S	N.A	
Iester (2000)	0.4% HY; 0.3% CMC	25 (28 eyes, 22 eyes)	N.A	N.A	N.A	Average of 2 scores: epithelial cell morphology and goblet cell density (3 points each)
Nelson (1988)	0.1% HY; 1.4% PVA	35 (20, 15)	1.4% PVA	N.S	0.2 vs -0.1	
Nelson (1988)	0.1% HY; 1.4% PVA	35 (20, 15)	HY	N.S	-0.3 vs 0	

Supplementary Table S7. Summary of Results of Studies, by Outcome Measure (Conjunctival Impression with Cytology). HY= Hyaluronic Acid; PVA= Polyvinyl Alcohol.

Visual Acuity						
Study (Year)	Study Arms	Number of Patients Analyzed for Outcome Measure (Treatment, Control)	Superior Eyedrop	P value	Difference in Treatment Effect (HY vs. Control) / Effect Magnitude (HY vs. Control) / Test for Treatment Effect (95% CI)	Remarks
Baeyens (2012)	0.18% HY; Normal Saline	195 (99, 96)	N.A	N.A	N.A	
Baeyens (2012)	0.18% HY; 0.3% Carbomer	190 (99, 91)	N.A	N.A	N.A	
Benelli (2010)	0.2% HY; 0.5% CMC	40 (20, 20)	HY	0.5895	0.1 (0.2) vs. 0.0 (0.1)	LOGMAR BCVA at distance of 3m measured
Benelli (2010)	0.2% HY; 0.18% HP Guar	40 (20, 20)	HY	0.2138	0.1 (0.2) vs. 0.0 (0.0)	LOGMAR BCVA at distance of 3m measured
Nelson (1988)	0.1% HY; 1.4% PVA	35 (20, 15)	N.A	N.A	N.A	Snellen's measured
Limberg (1987)	0.1% HY; 1% PVA	20	N.A	N.A	N.A	"BCVA and pinhole VA" measured

Supplementary Table S8. Summary of Results of Studies, by Outcome Measure (Visual Acuity). HY= Hyaluronic Acid; PVA= Polyvinyl Alcohol; CMC= Carboxymethylcellulose; BCVA= Best Corrected Visual Acuity.

Tear Meniscus						
Study (Year)	Study Arms	Number of Patients Analyzed for Outcome Measure (Treatment, Control)	Superior Eyedrop	P value	Difference in Treatment Effect (HY vs. Control) / Effect Magnitude (HY vs. Control) / Test for Treatment Effect (95% CI)	Remarks
MacDonald (2002)	0.1% HY; 1.4% PVA	32	Nil	N.A	0	Measured as either <0.2mm, 0.2-0.4mm or >0.4mm
Laflamme (1988)	0.1% HY; 1.4% PVA	12	1.4% PVA	N.A	0.25 vs. 0.33	Measured from scale of 0 (absent) to 3 (normal)
Limberg (1987)	0.1% HY; 1% PVA	20	N.A	N.A	N.A	

Supplementary Table S9. Summary of Results of Studies, by Outcome Measure (Tear Meniscus). HY= Hyaluronic Acid; PVA= Polyvinyl Alcohol

NITBUT						
Study (Year)	Study Arms	Number of Patients Analyzed for Outcome Measure (Treatment, Control)	Superior Eyedrop	P value	Difference in Treatment Effect (HY vs. Control) / Effect Magnitude (HY vs. Control) / Test for Treatment Effect (95% CI)	Remarks
McCann (2012)	0.15% HY ; CMC	49 (24, 25)	N.A	N.A	N.A	
Johnson (2008)	0.1% HY ; 0.3% Carbomer	65 (32, 33)	Carbomer	0.59	(-0.72, 1.25)	Measured in seconds

Supplementary Table S10. Summary of Results of Studies, by Outcome Measure (NITBUT). NITBUT= Noninvasive Tear Break-up Time; HY= Hyaluronic Acid; CMC= Carboxymethylcellulose.

Corneal Topography						
Study (Year)	Study Arms	Number of Patients Analyzed for Outcome Measure (Treatment, Control)	Superior Eyedrop	P value	Difference in Treatment Effect (HY vs. Control) / Effect Magnitude (HY vs. Control) / Test for Treatment Effect (95% CI)	Remarks
Benelli (2010)	0.2% HY; 0.5% CMC	40 (20, 20)	CMC	0.9999	-0.24 (-0.38, -0.11)(SD 0.29) vs -0.25 (-0.39, -0.10)(SD 0.30)	Measured wavefront aberrometry (in RMS values) - negative values signify improvement
Brignole (2005)	0.18% HY ; 1% CMC	21 (10, 11)	HY	>0.05	-0.01 vs. -0.07	Measured Surface Regularity Index - the higher the number, the better

Supplementary Table S11. Summary of Results of Studies by Outcome measures (Corneal Topography). HY= Hyaluronic Acid; CMC= Carboxymethylcellulose; RMS= Root Mean Square
